# Government policy interventions to reduce human antimicrobial use: A systematic review and evidence map

**DOI:** 10.1371/journal.pmed.1002819

**Published:** 2019-06-11

**Authors:** Susan Rogers Van Katwyk, Jeremy M. Grimshaw, Miriam Nkangu, Ranjana Nagi, Marc Mendelson, Monica Taljaard, Steven J. Hoffman

**Affiliations:** 1 School of Epidemiology and Public Health, University of Ottawa, Ottawa, Ontario, Canada; 2 Global Strategy Lab, Dahdaleh Institute for Global Health Research, Faculty of Health and Osgoode Hall Law School, York University, Toronto, Ontario, Canada; 3 Clinical Epidemiology Program, Ottawa Hospital Research Institute, Ottawa, Ontario, Canada; 4 Department of Medicine, University of Ottawa, Ottawa, Ontario, Canada; 5 Division of Infectious Diseases and HIV Medicine, Groote Schuur Hospital, University of Cape Town, Cape Town, South Africa; 6 Department of Health Research Methods, Evidence, and Impact, and McMaster Health Forum, McMaster University, Hamilton, Ontario, Canada; 7 Department of Global Health & Population, Harvard T.H. Chan School of Public Health, Harvard University, Boston, Massachusetts, United States of America; University of Connecticut Health Center, UNITED STATES

## Abstract

**Background:**

Growing political attention to antimicrobial resistance (AMR) offers a rare opportunity for achieving meaningful action. Many governments have developed national AMR action plans, but most have not yet implemented policy interventions to reduce antimicrobial overuse. A systematic evidence map can support governments in making evidence-informed decisions about implementing programs to reduce AMR, by identifying, describing, and assessing the full range of evaluated government policy options to reduce antimicrobial use in humans.

**Methods and findings:**

Seven databases were searched from inception to January 28, 2019, (MEDLINE, CINAHL, EMBASE, PAIS Index, Cochrane Central Register of Controlled Trials, Web of Science, and PubMed). We identified studies that (1) clearly described a government policy intervention aimed at reducing human antimicrobial use, and (2) applied a quantitative design to measure the impact. We found 69 unique evaluations of government policy interventions carried out across 4 of the 6 WHO regions. These evaluations included randomized controlled trials (*n =* 4), non-randomized controlled trials (*n =* 3), controlled before-and-after designs (*n =* 7), interrupted time series designs (*n =* 25), uncontrolled before-and-after designs (*n =* 18), descriptive designs (*n =* 10), and cohort designs (*n =* 2). From these we identified 17 unique policy options for governments to reduce the human use of antimicrobials. Many studies evaluated public awareness campaigns (*n =* 17) and antimicrobial guidelines (*n =* 13); however, others offered different policy options such as professional regulation, restricted reimbursement, pay for performance, and prescription requirements. Identifying these policies can inform the development of future policies and evaluations in different contexts and health systems. Limitations of our study include the possible omission of unpublished initiatives, and that policies not evaluated with respect to antimicrobial use have not been captured in this review.

**Conclusions:**

To our knowledge this is the first study to provide policy makers with synthesized evidence on specific government policy interventions addressing AMR. In the future, governments should ensure that AMR policy interventions are evaluated using rigorous study designs and that study results are published.

**Protocol registration:**

PROSPERO CRD42017067514.

## Abstract 2

In a systematic review, Susan Rogers Van Katwyk and colleagues assess policy interventions intended to reduce antimicrobial overuse.

## Author summary

**Why was this study done?:**

- Despite global commitments to reduce antimicrobial resistance and protect the effectiveness of antimicrobials, most countries have not yet started implementing government policies to reduce their overuse and misuse of antimicrobials.
- To the best of our knowledge, no evidence syntheses have attempted to identify the policy options available to government policy makers to tackle antimicrobial resistance by reducing antimicrobial use in humans.

**What did the researchers do and find?:**

- We searched 7 academic databases to identify impact evaluations of government policy interventions aiming to reduce human antimicrobial use that were published in any language before January 28, 2019.
- We found 69 studies that evaluated government policy interventions to reduce antimicrobial use around the world. From these, we were able to describe 17 different types of policies that governments have used to tackle this major driver of antimicrobial resistance in humans.
- Commonly used policy strategies included public awareness campaigns and antimicrobial guidelines; however, other policy strategies focused on vaccination, stewardship, and changing regulations around prescribing and reimbursement.
- We found 4 randomized controlled trials and 35 studies using rigorous quasi-experimental designs. The remaining 30 studies used uncontrolled and descriptive study designs.

**What do these findings mean?:**

- Our systematic evidence map suggests that governments have a variety of policy options at their disposal to respond to the growing threat of antimicrobial resistance.
- Unfortunately, most existing policy options have not been rigorously evaluated, which limits their usefulness in planning future policy interventions.
- To avoid wasting public resources, governments should ensure that future antimicrobial resistance policy interventions are evaluated using rigorous study designs, and that study results are published.

## Introduction

Antimicrobial resistance (AMR) is currently high on the global political agenda. This attention has opened a rare policy window for achieving meaningful action on AMR [1–7]. Although the potential for AMR has been recognized since the earliest days of antibiotics [8], the misuse and overuse of antimicrobials has persisted over decades, contributing to the development of resistance [9]. AMR is now expected to have severe consequences for human health, social well-being, and economic development. AMR has already rendered some infections untreatable using existing antimicrobials [10,11], and global projections suggest that AMR could derail the Sustainable Development Goals, driving an estimated 24 million people into extreme poverty and exacerbating global economic inequality [12], and potentially resulting in tens of millions of deaths [13].

Successfully overcoming the threat posed by AMR will require multi-sectoral and multi-jurisdictional cooperation to protect the effectiveness of existing and future antimicrobials [2,3,5]. Recent political initiatives addressing AMR, including the 2016 United Nations resolution [14] and the 2017 Berlin Declaration of the G20 Health Ministers [15], are promising signs that governments and international agencies are mobilizing to act on AMR. The 194 member states of the World Health Organization (WHO) agreed to develop national AMR action plans by 2017 [16], and countries have largely responded [17].

Despite these positive steps, most countries have not yet started implementing policies to reduce their overuse and misuse of antimicrobials [17]. Evidence from high-income countries suggests that reducing antimicrobial use is associated with lower rates of resistance [9], yet there is limited evidence on what types of government policy interventions effectively reduce antimicrobial use. Typically, government policy changes are useful tools for improving public health when the health threat requires widespread change and uniform compliance with a set of minimum standards [18]. However, research on AMR has principally focused on changing the prescribing behaviours of individual physicians [19], rather than creating large-scale reductions in antimicrobial use through population-wide interventions.

Given that governments are currently grappling with the challenge of implementing AMR policies under their recently developed national action plans, a focus on the potential impact of government policy interventions on antimicrobial use is timely. Governments are currently attempting to weigh the merits of numerous types of policy interventions that could safely reduce antimicrobial use, utilizing policy levers such as legislation, taxation, economic incentives, funding support, public awareness campaigns, and regulation of professionals and businesses whose work might affect AMR [18]. Policy makers would benefit from a tool that catalogues and assesses the government policy responses that have been used in various contexts and health system settings. Thus we undertook a systematic evidence mapping project to support evidence-informed action on AMR at the government level, by identifying, describing, and assessing the full range of government policy interventions aiming to reduce human antimicrobial use that have been implemented and evaluated.

## Methods

A protocol describing the full methods of this project was published in advance [20] and registered in PROSPERO (CRD42017067514). Deviations from the protocol are noted below, and the paper has been reported in accordance with the Preferred Reporting Items for Systematic Review and Meta-Analysis (PRISMA) guidelines [21]. In brief, we produced an evidence map that identifies government policy interventions aiming to reduce antimicrobial use in humans. To be included in the evidence map, studies had to (1) clearly describe a government policy intervention aiming to reduce human antimicrobial use and (2) apply a quantitative design to measure the impact.

### Search strategy and selection criteria

We searched 7 electronic databases from medicine and the social sciences (MEDLINE, CINAHL, EMBASE, PAIS Index, Cochrane Central Register of Controlled Trials, Web of Science, and PubMed [articles not indexed in MEDLINE]) from inception to January 28, 2019, without language or date limits. Targeted web searching was used to identify grey literature, and the ProQuest Dissertations & Theses database was used to identify dissertations. We contacted subject-matter experts in each of WHO’s 6 regions to identify missing studies.

We screened titles and abstracts against 3 inclusion criteria: (1) the evaluated intervention was a policy intervention defined as an intervention enacted by a government or government agency at the federal, state, provincial, or municipal level that aimed to change antimicrobial use through education, restriction, incentivization, coercion, training, persuasion, changing the physical or social context, modelling appropriate behaviour, or reducing barriers to action [22]; (2) the study quantitatively evaluated the effect of the intervention; and (3) the study assessed an outcome measure related to human antimicrobial use such as consumption, dosing, prescribing, or sales of an antibiotic, antiviral, antiparasitic, or antifungal drug. Examples of interventions include regulating the sales of antimicrobials, restricting the use of last-resort antibiotics, and launching public awareness campaigns. Titles and abstracts were each independently screened by 2 reviewers (SRVK and S Jones, A Srivastava, or RN), and disagreements were resolved by consensus. The full text of potentially relevant studies was screened by 2 reviewers (SRVK and MN or RN). Non-English articles were translated using Google Translate, or a translation was requested from the corresponding author.

### Data analysis

Data on study characteristics, study participants, interventions, analyses, and measured effects were extracted in duplicate by 2 reviewers (SRVK and MN or RN) using a customized data extraction tool (see S2 Text). In consultation with SJH and JMG, SRVK grouped studies according to the Behaviour Change Wheel framework’s intervention functions and our definition of policy intervention [22]. Where appropriate, studies were coded with multiple Behaviour Change Wheel intervention functions; however, studies were coded with a single Behaviour Change Wheel policy approach. We inductively identified and described policy options based on groupings of similar interventions, and coded studies according to their region, study design, and the intervention functions of the Behaviour Change Wheel. WHO regions were used to group countries; the region of the Americas was subdivided into Canada/US and Latin America.

## Results

**Fig 1:**
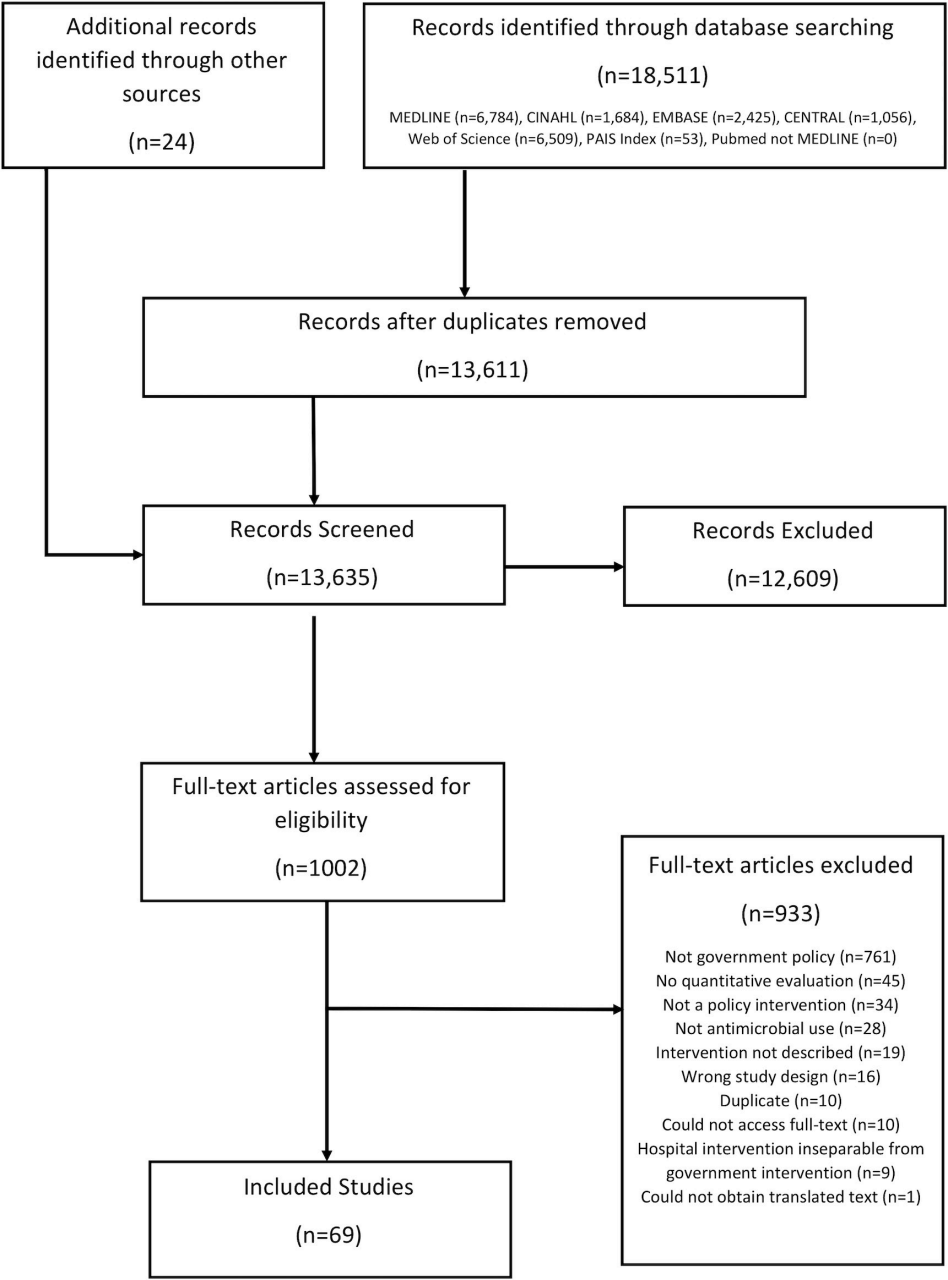
PRISMA summary flow chart. CENTRAL, Cochrane Central Register of Controlled Trials.

From 13,635 abstracts, we identified 69 evaluations of government policy interventions to reduce human antimicrobial use. Fig 1 shows the full summary of screening and inclusion. Of the 69 included studies, 67 focused on antibiotics and 2 on antimalarials; no studies aimed to reduce the use of other antimicrobial agents. The majority of policies targeted healthcare workers (*n =* 44) or healthcare workers and the community (*n =* 13), while the remaining 12 policies exclusively targeted a community audience. We found evaluations in 4 of the 6 WHO regions—the Americas (*n =* 24), Western Pacific (*n =* 22), Europe (*n =* 21), and Africa (*n =* 2)—but did not identify any evaluations from the South East Asian region or the Eastern Mediterranean region. Of the 69 included studies, 67 were published in English and 2 were published in Spanish.

Using our definition of policy intervention, we organized studies according to the policy categories of the Behaviour Change Wheel framework. Table 1 describes the interventions of the included studies. The largest grouping of policies was regulatory interventions (*n =* 27), followed by guidelines (*n =* 18), communication policies (*n =* 17), legislation (*n =* 3), and fiscal measures (*n =* 3). One evaluation was identified for service provision policies; however, we did not identify any social planning policies. Regulatory policies (*n =* 20/27) and legislation (*n =* 3/3) were largely organized at the national level. Communication policies were organized at different levels of government; 8 were at the national level, 3 were at the state/provincial level, and 6 were at the regional level (municipality, county, or other geographic unit). Similarly, 12 of the guidelines were at the national level, 2 were at the state/provincial level, and 4 were at the regional level. One of the fiscal measures policies was at the national level, and 2 were at the regional level. The sole service provision policy was organized at the national level.

**Table 1:** Included studies by policy approach.

Policy approach	Description*	Studies
Regulatory interventions	Establishing rules or principles of behaviour or practice	[23–49]
Guideline interventions	Creating documents that recommend or mandate practice	[50–67]
Communication interventions	Using print, electronic, telephonic, or broadcast media	[68–84]
Legislation interventions	Making or changing laws	[85–87]
Service provision interventions	Delivering a service	[88]
Environmental/social planning interventions	Designing or controlling the physical or social environment	None
Fiscal interventions	Using the tax system and other financial measures to reduce or increase the financial cost	[89–91]

*Policy approach descriptions from the Behaviour Change Wheel framework [22].

The majority of the 69 included studies were retrospective evaluations using routinely collected data from health insurance databases or electronic health records (*n =* 46), or sales data from IMS Health (*n =* 14). Four of the included studies were randomized controlled trials, 3 were non-randomized controlled trials, 7 used non-randomized controlled before-and-after designs, 25 used time series designs, 18 used uncontrolled before-and-after designs, 10 used descriptive methods, and 2 used cohort study designs (Box 1). The included studies predominantly used antibiotic consumption measured as defined daily doses (*n =* 25) or physician prescribing rates (*n =* 26) as an outcome measure.

**Box 1. Definitions of included study designs**

**Randomized controlled trial:** An experimental study in which people are allocated to different interventions using methods that are random.

**Non-randomized controlled trial:** An experimental study in which people are allocated to different interventions using methods that are not random.

**Time series design:** A study that uses observations at multiple time points before and after an intervention. The design attempts to detect whether the intervention has had an effect significantly greater than any underlying trend over time.

**Non-randomized controlled before-and-after design:** A study in which observations are made before and after the implementation of an intervention, both in a group that receives the intervention and in a control group that does not.

**Uncontrolled before-and-after design:** A study in which observations are made before and after the implementation of an intervention in a single intervention group.

**Cohort design:** A study in which designated groups of people are followed over time to ascertain the occurrence of an event.

**Descriptive design:** A study that employs observational, cross-sectional, ecological, or other descriptive methods, to infer or hypothesize the impact of an intervention.

All definitions except “cohort design” and “descriptive design” were taken from Cochrane’s Effective Practice and Organisation of Care Group [92].

Among the 69 included evaluations, we identified 17 distinct policy options that have been evaluated for their ability to reduce antimicrobial use. Table 2 summarizes these policy options and lists the studies that evaluated specific manifestations of them. By far the most common of these policy options were informational strategies, including public awareness campaigns (*n =* 17), which informed healthcare workers and/or the public about AMR and antimicrobial overuse, and antimicrobial guidelines (*n =* 13), which provided information to healthcare workers on the preferred use of antimicrobial drugs or preferred treatments for resistant infections. These strategies were widely used across most regions, and in particular represented a large proportion of the interventions evaluated in Canada/US [52,53,59,61,62,69,74,76–78,80,81] and Europe [63,65,67,68,70,71,73,75,79,82,90,91].

**Table 2:** Description of policy options that have aimed to reduce human antimicrobial consumption.

Policy option	Description	Studies
Policies to improve infection prevention and stewardship efforts		
Published antimicrobial guidelines	Information provided to healthcare workers on the preferred use of antimicrobial drugs, or preferred treatment for resistant infections	[50–53,55,57–64]
Vaccination guidelines	Guidelines and policies recommending vaccinations likely to reduce antimicrobial use	[90]
Committee development	Guidelines encouraging the formation of expert groups on stewardship and resistance	[56]
Stewardship	A requirement that specific stewardship policies be introduced	[27,33,65]
Disclosure	A requirement for public disclosure of antibiotic use level	[32]
Funding	Provision of funding towards a specific stewardship program or goal	[88]
Policies to educate health professionals, policy makers, and the public on sustainable antibiotic use		
Public awareness	Public educational campaigns drawing on media and internet to inform healthcare workers and/or the public about antimicrobial resistance	[68–84]
Feedback	Audit and feedback to providers about their antimicrobial use habits	[54,66]
Policies to change incentives that encourage antibiotic overuse and misuse		
Reimbursement penalty for patients	A reduction in the amount that a patient is reimbursed for a prescription by a drug plan	[41]
Reimbursement penalty for prescribers	The prescriber is not paid for their services unless the guidelines for prescribing antimicrobials are met	[26]
Restricted reimbursement	Introduces an additional step in the prescribing pathway such as consultation with a specialist or provision of proof of infection in order for the prescription to be reimbursed	[30,31,34,36,38]
Restricted use	Introduces an additional step in the prescribing pathway such as consultation with a specialist or provision of proof of infection in order for the prescription to be dispensed	[23,28,35,43,47,48]
Pay for performance	Pay-for-performance funding provided to healthcare centres that meet particular antimicrobial-use-related guidelines and targets	[67,89,91]
Policies to change features of the health system		
Professional regulation	Changes to codes of practice with regards to what can be done by members of different healthcare professions	[87]
Prescription requirement	Requirement of a prescription to purchase antimicrobial drugs	[24,25,39,40,44,85,86]
Formulary change	Removal of a drug from the formulary or addition of a drug to the formulary	[37]
National essential medicines policies	Introduction of policies in line with WHO’s essential medicines policies	[29,45,46,49]

**Fig 2:**
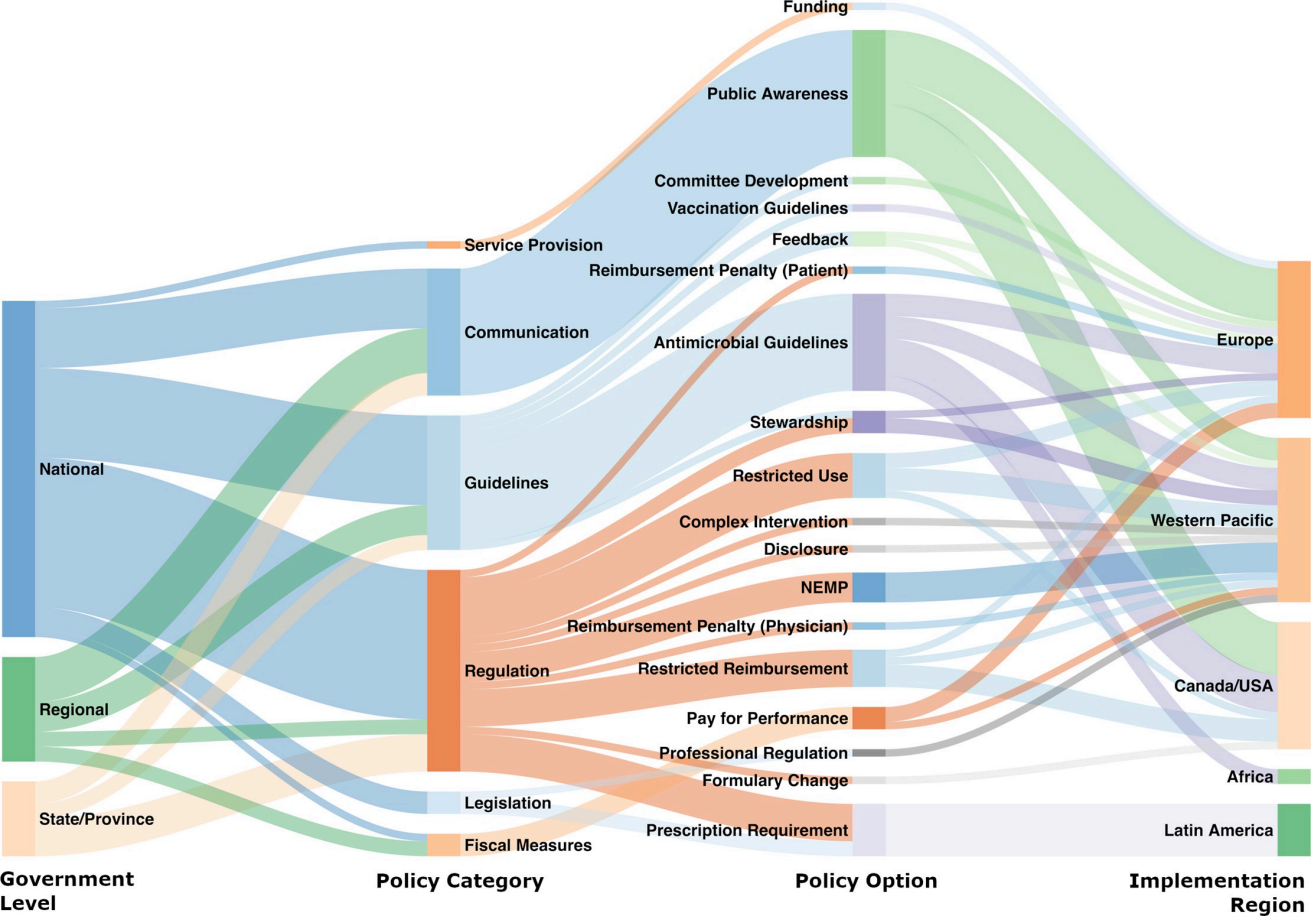
Evidence map of the relationships between government level, policy approach (policy category), policy option, and region of implementation. The thickness of lines represents the proportions of included studies. The colors show the grouping of policies and trace the flow of policies between categories. NEMP, national essential medicines policy.

Other policy options were less commonly reported and tended to group regionally, as can be seen in Fig 2. For example, 7 studies were categorized as “prescription requirement” policies, which were regulatory and legislative policies essentially banning the sale of over-the-counter antibiotics by requiring a prescription from a healthcare professional [24,25,39,40,44,85,86]. These policies were implemented starting in the late 1990s in countries or regions in Latin America where over-the-counter antibiotic sales were not previously prohibited, or where existing regulations were not enforced. Countries in WHO’s Western Pacific region tried a diverse range of strategies, most of which were evaluated only once or twice. These policies were largely implemented in China [27,29,33,42,43,45–49,60,66,89], South Korea [32,49,57,87], and Taiwan [26,30], and included disclosure requirements for hospitals to post their antibiotic use rates online, professional regulation strategies that changed the codes of practice around the prescribing and dispensing of antibiotics by different health professions, and reimbursement penalties for physicians, who were not paid for their services unless their prescriptions met the guidelines for antibiotic prescribing. Along similar lines, 1 European country (Denmark) explored using reimbursement penalties targeting patients, where the national health insurance plan reimbursed patients a smaller proportion of the antibiotic cost than was previously reimbursed [41]. Three studies in Canada/US [34,36,38] and 1 each in Europe [31] and the Western Pacific [30] also tried reimbursement restrictions, where the patient was not reimbursed the cost of an antibiotic by their national health insurance plan unless the physician met particular guidelines such as proving the existence of an infection or consulting with an infectious disease specialist. In the African region, we found only 2 studies, both targeting the use of antimalarial drugs, and both employing published guidelines to change prescribing [50,51]. Some national-level interventions were evaluated in multiple studies using different methods, populations, and evaluation time frames; these included the ban on over-the-counter sales of antimicrobials in Chile [24,25,44], the Antibiotics Are Not Automatic campaign in France [70,71,73,82], and the national essential medicines policy in China [29,45,46,49].

## Discussion

### Principal findings

Around the world, governments are currently working to develop policy responses to the growing threat of AMR. In our evidence map, we identified 69 evaluation studies looking at the impact of policy interventions on antimicrobial use across 4 of WHO’s 6 regions. From this search, we were able to identify 17 different policy options and examples of each that governments can use to inform their future AMR policies.

Many of the policy options identified in this map were evaluated in only a few studies. These evaluations were highly regionalized, which likely results from similarities between contexts and health systems within regions of a country, or in neighbouring countries. Policy makers in other parts of the world who operate with similar contextual problems or health systems may find these policies useful models for policy development in their countries. For example, the prescription requirement policies [24,25,39,40,44,85,86] were implemented in 5 Latin American countries where over-the-counter antibiotic sales were formally or informally permitted. While this type of policy would not be useful in Canada and the US, where prescriptions are already required, the regulations and legislation used in Latin America may be a useful model for many other countries, such as those in Africa, the Eastern Mediterranean, South East Asia, and the Western Pacific that currently allow over-the-counter sales of antibiotics, and where overuse of antimicrobials is likely to decrease in response to this restriction.

Similarly, we identified several policies that used electronic medical records and national health insurance systems to change physician and patient behaviours around antimicrobial use. These policies, including restricted reimbursements and reimbursement penalties for patients and prescribers, were used in high-income jurisdictions such as Canada, Sweden, and Taiwan. These policies take a different approach, targeting overuse through restrictive and coercive financial mechanisms.

### Policy implications

Given the complexity of AMR, and the need to balance conservation of antimicrobial effectiveness with ensuring access to appropriate antimicrobials for those who need them [2], there is unlikely to be a “silver bullet” intervention that solves the global AMR problem. These 17 government policy interventions offer a starting point for countries to adapt to their local context. Since most of these 17 policies have been evaluated only once or twice and in particular contexts, it would be unwise to draw strong conclusions about their effectiveness. Indeed, many of these interventions were evaluated using low-quality, non-randomized designs; while many systematic reviews would exclude studies on this basis, we retained them in our evidence map to ensure that we captured the widest range of policy options possible. To avoid future waste of public resources, and in line with WHO recommendations for national action on AMR [93], governments should ensure that AMR policy interventions are evaluated using rigorous study designs and that study results are published.

Not surprisingly, our evidence map found that public awareness campaigns and guidelines were commonly used strategies for reducing antimicrobial use across all regions. These educational approaches are traditional public health strategies and have been promoted by both WHO and the UK’s Review on Antimicrobial Resistance [13]. While launched at the government level, many of these programs and policies still focus on changing the practice of individual prescribers, usually physicians, rather than targeting other healthcare professionals or altering healthcare structures to reduce overuse and misuse of antibiotics. Different governments have different policy levers at their disposal, including the ability to implement complex regulatory, legislative, fiscal, and service provision policies, which could potentially bring about more dramatic change than policies focused on individual prescriber behaviour change. Many approaches to reducing antimicrobial consumption can only be implemented by governments, including many of the policies we identified (e.g., professional regulation, restricted reimbursement, and prescription requirements) as well as policies identified by others in academic literature for which we did not find any evaluations (e.g., creating human-only classes of antimicrobials [1], banning direct-to-consumer advertising [3,6], and using tax or fiscal measures [94]). Given that governments can employ a broad range of policy options beyond public awareness campaigns and guidelines, the full range of possible AMR policies should be further explored.

### Strengths and limitations

Our evidence map represents the first systematic effort, to our knowledge, to identify government policy interventions and specific policy mechanisms for reducing human antimicrobial use. We worked with 3 research librarians from 3 disciplines and contacted experts around the world to identify published and grey literature on government and AMR policies. However, we recognize that there are implemented policies (e.g., [95]) that have not been captured in this evidence map. We suspect that these studies have not been evaluated, or have not been evaluated with respect to antimicrobial use, or the results of these studies have not been made public.

As with many studies about AMR, we were unable to directly investigate the human health impact of government action on AMR due to the complex relationships among AMR, the use of antimicrobials in humans, animals, and agriculture, and health outcomes. This complexity will continue to be a challenge for AMR research until such a time as “one health” monitoring systems for both antimicrobial use and AMR improve. However, reductions in antimicrobial use are a more immediate measure of policy impact, and large-scale reductions in antimicrobial use are likely to lead to lower levels of resistance [96]. Reducing antimicrobial use is therefore a valuable target for policy makers tackling AMR at the population level.

### Conclusions

Our identification of 17 different policy strategies for reducing human antimicrobial use suggests that governments have a variety of policy options at their disposal for mitigating AMR. However, we also note that most existing policy options have not been rigorously evaluated, and some commonly discussed policy options have not been evaluated for their impact on antimicrobial use. To avoid wasting public resources, governments should ensure that future AMR policy interventions are evaluated using rigorous study designs and that study results are published.

## Supporting information

S1 PRISMA Checklist (DOCX)

S1 Table Intervention and study design summary of all included studies. (DOCX)

S1 Text Search strategies. (DOCX)

S2 Text Data extraction tool. (DOCX)

## Abbreviations

AMR: antimicrobial resistance
WHO: World Health Organization
