# Supplementary material for: Government policy interventions to reduce human antimicrobial use: A systematic review and evidence map
Source: PLoS Med. 2019 Jun 11;16(6):e1002819. doi: 10.1371/journal.pmed.1002819 (PMC6559631; doi:10.1371/journal.pmed.1002819)
Supplement: S1 Table — (DOCX) [file pmed.1002819.s002.docx]

| Study | Country | Policy Level | Enacting Government | Intervention Summary | Methods | Outcome | Policy Strategy | Policy Option | BCW Intervention Function | Impact | Target Audience |
| --- | --- | --- | --- | --- | --- | --- | --- | --- | --- | --- | --- |
| D'Acremont V, et al. Malaria Journal. 2011;10:107. | Tanzania | Regional | City Medical Office and Municipal Health Department | Provision of guidelines and education for use of anti-malarial drugs with rapid malaria diagnostic testing. | Cluster RCT using routinely collected data. | Monthly number of anti-malarial or antibiotic doses issued | Guidelines | Antimicrobial Guidelines | Education | Expected | Health Care Worker^[[1]](#footnote-1)^ |
| Finkelstein JA, et al. Pediatrics. 2008;121(1):e15-23. | United States of America | Regional | Massachusetts Department of Public Health | Annual public and provider education campaign "REACH Mass" during cold and influenza season. | Cluster RCT using health insurance claims data. | Number of oral antibiotic dispensed per person-year of observation | Communication | Public Awareness | Education | Expected | Community; Health Care Worker |
| Hallsworth M, et al. Lancet. 2016;387(10029):1743-52. | England | National | Department of Health – Chief Medical Officer | Feedback letter from England’s Chief Medical Officer informing high using practitioners that they were prescribing antibiotics at a higher rate than 80% of practices in their region; and provider education materials | A 2x2 factorial RCT using national prescribing data. | Prescriptions per 1000 STAR-PU | Guidelines | Feedback | Education; Persuasion | Expected | Health Care Worker |
| Yip W, et al. Health affairs. 2014;33(3):502-10. | China | Regional | Government of Ningxia | Pilot program changing fee-for-services to pay-for-performance in rural healthcare settings. | Cluster RCT using data from health centre information system, a household survey, a village clinic survey and a township health centre survey. | Prescription Rate | Fiscal Measures | Pay for Performance | Environmental restructuring; | Expected | Health Care Worker |
| Bauraind I, et al. JAMA. 2004;292(20):2468-70. | Belgium | National | Belgian Scientific Institute of Public Health | Two public campaigns using booklets, handouts, posters and television to promote rational use of antibiotics and reduce overuse and misuse in the community. | Interrupted time-series analysis using IMS Health data. | DDD | Communication | Public Awareness | Education | Expected | Community |
| Bernier A, et al. Antimicrobial Agents and Chemotherapy. 2014;58(1):71-7. | France | National | French National Health Insurance | National public health campaign “Antibiotics Are Not Automatic!” repeated every winter. | Interrupted time-series analysis using data from the French National Health Insurance Database. | Outpatient antibiotics prescribed and reimbursed per 1000 inhabitants | Communication | Public Awareness | Education | Expected | Community |
| Dommergues MA, Hentgen V. Scandinavian Journal of Infectious Diseases. 2012;44(7):495-501. | France | National | French National Health Insurance | National public health campaign “Antibiotics Are Not Automatic!” repeated every winter. | Time-series analysis using data from a permanent survey of medical prescriptions. | Prescription Rate | Communication | Public Awareness | Education | Expected | Community |
| Dowell D, et al. American Journal of Public Health. 2012;102(1):148-55. | United States of America | Regional | Centers for Disease Control and Prevention | Publication and circulation of revised CDC guidelines for the treatment of gonorrhea through MMWR, state and local health departments, a dear colleague letter, and a national press conference. | Interrupted time-series using data from a national network of health departments conducting enhanced STD surveillance. | Percentage of Infections treated with antibiotic | Guidelines | Antimicrobial Guidelines | Education | Expected | Health Care Worker |
| Hernandez-Santiago V, et al. Journal of Antimicrobial Chemotherapy. 2015;70(8):2397-404. | Scotland | Regional | National Health Service Tayside | National policy changes including guidelines, educational interventions and feedback for practices, and information leaflets to explain prescribing changes to individuals. | Interrupted time-series analysis using administrative data. | Quarterly rate of 4C antibiotics dispensed per 1000 patients | Guidelines | Antimicrobial Guidelines | Education; Persuasion; | Expected | Health Care Worker |
| Mamdani M, et al. American Journal of Medicine. 2007;120(10):893-900. | Canada | State/  Province | Government of Ontario | Formulary change to restrict the use of fluoroquinolones requiring prescribers to indicate the reason for fluoroquinolone use. | Time series analysis using administrative healthcare databases. | Prescriptions per 1000 persons/quarter | Regulation | Restricted Use | Restriction | Expected | Health Care Worker |
| Manns B, et al. BMC Health Services Research. 2012;12:290. | Canada | State/  Province | Alberta Health and Wellness | Regulation requiring physicians to submit additional paperwork in order for patients to have their quinolone prescription reimbursed unless they registered as a designated quinolone prescriber. | Interrupted time-series analysis using administrative drug claims data. | Prescription Rate | Regulation | Restricted Reimbursement | Restriction; Coercion | Neutral | Health Care Worker |
| Marra F, et al. Journal of Antimicrobial Chemotherapy. 2005;55(1):95-101. | Canada | State/  Province | British Columbia Government | Formulary change delisting and later relisting clarithromycin, and adding valaciclovir and famciclovir to the formulary. | Interrupted time series using BC PharmaNet data. | DDDs per 1000 population/day | Regulation | Formulary Change | Restriction; | Expected | Health Care Worker |
| Marshall D, et al. Journal of Health Services Research & Policy. 2006;11(1):13-20. | Canada | State/  Province | Ontario Ministry of Health and Long-term Care | Limitation on reimbursement of two fluoroquinolones to patients for whom second line antibiotics were indicated, while other patients would be reimbursed for first-line drugs only. | Interrupted time-series analysis using data from Bayer Health Inc. | Prescribing volume | Regulation | Restricted Reimbursement | Restriction; | Opposite | Health Care Worker |
| Sabuncu E, et al. Plos Medicine. 2009;6(6). | France | National | French National Health Insurance | Nationwide public health campaign “Antibiotics Are Not Automatic!” targeting the general public and health care professionals. | Interrupted time series analysis using administrative data from the national health insurance programme. | Weekly rate of antibiotic prescriptions per 100 inhabitants | Communication | Public Awareness | Education | Expected | Community; Health Care Worker |
| Sun J, et al. Journal of Global Antimicrobial Resistance. 2015;3(2):95-102. | China | National | Ministry of Health China | Multifaceted national intervention to promote rational use of antibiotics including target setting, monitoring, prescribing restrictions, training and revoking prescribing rights for prescribers who continuously do not meet the hospital's targets. | Interrupted time-series using data from the National Antimicrobial Clinical Use and Resistance Monitoring Network. | Prescription Rate | Regulation | Complex Intervention | Coercion; Restriction; Environmental Restructuring; Education; Training | Expected | Health Care Worker |
| Weiss K, et al. Clinical Infectious Diseases. 2011;53(5):433-9. | Canada | State/  Province | Quebec Government | Provincial educational program provided graphic guidelines on antibiotic use for common conditions in outpatient settings which were sent to all physicians and pharmacists, accompanied by a letter from key provincial stakeholders. | Interrupted time series using IMS Health data. | Number of antibiotic prescriptions per 1000 inhabitants | Guidelines | Antimicrobial Guidelines | Education; Persuasion; | Expected | Health Care Worker |
| Wutzke SE, et al. Health promotion international. 2007;22(1):53-64. | Australia | National | NPS is an independent, non-profit organization funded by the Australian Government Department of Health and Ageing | Educational intervention for patients and health professionals: ‘Common colds need common sense’ using TV, radio and magazines. | Interrupted time series using consumer reported use of antibiotics for colds and flu, and data from Medicare Australia and the Drug Utilization Sub-Committee (DUSC) of the Pharmaceutical Benefits Advisory Committee. | Median original scripts/1000 consultations/GP/year | Communication | Public Awareness | Education | Expected | Community; Health Care Worker |
| Bou-Antoun S, et al. Journal of Antimicrobial Chemotherapy. 2018;73(10):2883-92. | England | National | NHS England | NHS England Quality Premium: Financial incentives for Clinical Commissioning Groups to reduce antibiotic prescribing in primary care | Interrupted time series analysis using data from the Clinical Practice Research Datalink. | Antibiotic items per 1000 RTI consultations | Fiscal Measures | Pay for Performance | Incentivisation; | Expected | Health Care Worker |
| Ellegard LM, Dietrichson J, Anell A. Health Economics. 2018;27(1):e39-e54. | Sweden | Regional | County Health Authorities in Sweden | Pay-for-performance (P4P), monetary incentives to reach predefined targets in antibiotic prescribing | Difference-in-differences (DID) model using data from the Swedish Prescribed Drug Register | PcV share: the number of narrow-spectrum penicillin V prescriptions divided by the total number of RTI antibiotics prescriptions to children between 0 and 6 years of age. | Fiscal Measures | Pay for Performance | Incentivisation; | Expected | Health Care Worker |
| Kelly AA, et al. A Infection control and hospital epidemiology. 2017;38(5):513-20. | USA | National | Department of Veterans Affairs (VA) Veterans Health Administration | A Veterans Health Administration National Antimicrobial Stewardship Initiative including continuing education, disease-specific guidelines, and development of example policies. | Interrupted time series analysis using resource utilization data from the corporate data warehouse. | Days of therapy per 1,000 in patient bed days of care | Guidelines | Antimicrobial Guidelines | Education; Enablement; Modelling | Expected | Health Care Worker |
| Ouldali N, et al. Clinical infectious diseases. 2017;65(9):1469-76. | France | National | French Agency for the safety of Health Products | Introduction of national guidelines (2011) on antibiotic prescriptions for acute respiratory tract infection in pediatric emergency departments. | Interrupted time series analysis using routinely collected hospital data. | Antibiotic prescribtion rate for ARI per 1000 PED visits | Guidelines | Antimicrobial Guidelines | Education; | Expected | Health Care Worker |
| Tang YQ, et al. Cost Effectiveness and Resource Allocation. 2018;16. | China | National | Government of China | Chinese government issued administrative rules for the clinical use of antibiotics which included the creation of restricted groups of antibiotics and penalties for violating the administrative rules. | Controlled ITS with a difference-in-difference approach | Volume of procured medicines in DDD | Regulation | Restricted Use | Coercion; Education; Restriction | Expected | Health Care Worker |
| Walker AJ, Curtis HJ, Goldacre B. Journal of Antimicrobial Chemotherapy 2019. | England | National | NHS England | Five year antimicrobial stewardship strategy and a suite of actions taken by the Chief Medical Officer | Interrupted time series analysis using the Prescription Cost Analysis Dataset from NHS Digital. | Prescriptions per 1000 STAR-PU | Guidelines | Stewardship | Environmental Restrcutring; Education | Expected | Health Care Worker |
| Wu J, et al. The Journal of international medical research. 2018;46(4):1326-38. | Australia | National | NPS Medicinewise Australia | NPS Medicinewise: a series of nationwide educational and advertising interventions for general practitioners and consumers. | Bayesian structural time series model using prescriber level data from PBS and the Medicare Benefit Schedule. | Total number of dispensed scripts | Communication | Public Awareness | Education | Expected | Health Care Worker |
| Zhen L, Jin C, Xu H-N. BMC health services research. 2018;18(1):777. | China | National | Huangdao Bureau of Health | Rural Clinics Prescription Commitment: creation of antibiotic guidelines with audit and feedback to rural prescribers. | Interrupted time series analysis using data from electronic medical records. | Total number of dispensed scripts | Guidelines | Feedback | Education; | Expected | Health Care Worker |
| Fuertes EI, et al. Canadian Journal of Public Health. 2010;101(4):304-8. | Canada | State/  Province | BC Centres for Disease Control; Provincial Government British Columbia | Provincial public education campaign "Do Bugs Need Drugs?" targeting school children and healthcare professionals. | Interrupted time-series analysis using data from BC PharmaNet. | DDD per 1000 population/day | Communication | Public Awareness | Education | Expected | Community; Health Care Worker |
| Santa-Ana-Tellez Y, et alPloS one. 2013;8(10):e75550. | Mexico and Brazil | National | Governments of Mexico and Brazil | Restriction on the sale of antibiotics without a prescription in pharmacies, and introduction of a fine on the owners of pharmacies for non-compliance. | Interrupted time-series analysis using IMS Health data. | DDD per 1000 inhabitants/day | Regulation | Prescription Requirement | Restriction; Coercion | Expected | Community; Health Care Worker |
| Santa-Ana-Tellez Y, et al. Antimicrobial agents and chemotherapy. 2015;59(1):105-10. | Mexico and Brazil | National | Governments of Mexico and Brazil | Restriction on the sale of antibiotics without a prescription in pharmacies, and introduction of a fine on the owners of pharmacies for non-compliance. | Interrupted time-series analysis using IMS Health data. | DDD per 1000 inhabitants/day | Regulation | Prescription Requirement | Restriction; Coercion | Expected | Community; Health Care Worker |
| Wirtz VJ, et al. Tropical Medicine & International Health. 2013;18(6):665-73. | Chile, Columbia, Mexico and Venezuela | Multiple Policies | Governments of Chile, Colombia, and Venezuela | Banning over-the-counter antibiotics sales and requiring retention of prescriptions and inspections of pharmacies. | Interrupted time-series using IMS Health data. | DDD per 1000 inhabitants/day | Regulation | Prescription Requirement | Restriction; Coercion; Education; | Expected | Community; Health Care Worker |
| Formoso G, et al. BMJ. 2013;347:f5391. | Italy | Regional | Emilia-Romagna Regional Agency for Health and Social Care | Provincial information campaign "Antibiotics, solution or problem" using social marketing and consumer research and communication techniques. | Controlled non-randomized trial using regional prescribing databases. | DDD per 1000 inhabitants/day | Communication | Public Awareness | Education | Expected | Community |
| Gonzales R, et al. Health Services Research. 2005;40(1):101-16. | United States of America | State/  Province | Colorado Department of Public Health and Environment | State public education campaign ‘‘Be S.M.A.R.T. about Antibiotics’’ with brochures, educational information, and posters. | A controlled non-randomized trial using administrative claims data. | Prescription Rate | Communication | Public Awareness | Education | Expected | Community |
| Hennessy TW, et al. Clinical Infectious Diseases. 2002;34(12):1543-50. | United States of America | Regional | Centers for Disease Control and Prevention | Public and provider education campaign including workshops for community-health aides and physicians, follow-up visits to the community-health aides to review the principals of appropriate use, and village-wide educational meetings. | Cluster non-randomized trial using individual medical records from village clinics. | Number of courses of antibiotics per respiratory infection visit | Communication | Public Awareness | Education; Training; | Expected | Community; Health Care Worker |
| Bastiaens GJH, et al. Malaria Journal. 2011;10:76. | Tanzania | Regional | Tanzanian Ministry of Health and Social Welfare | Pilot test of replacing microscopy with malaria rapid diagnostic testing and restricting treatment to RDT positive patients in order to tackle malaria over-diagnosis. | Controlled before and after study using questionnaire data. | Prescription Rate | Guidelines | Antimicrobial Guidelines | Restriction; Education; Training | Expected | Health Care Worker |
| Duval M, Desrosiers M. Otolaryngology-- Head and Neck Surgery. 2007;136(2):258-60. | Canada | State/  Province | Government of Quebec | Mailout of guidelines for acute bacterial rhinosinusitis along with information cards, supported by a press conference and media campaign. | Controlled before and after study using data from IMS Health. | Number of antibiotic prescriptions | Guidelines | Antimicrobial Guidelines | Education | Expected | Health Care Worker |
| Gong Y, et al. Health Policy and Planning. 2016;31(1):21. | China | National | Chinese Ministry of Health | Multifaceted National Chinese Essential Medicines Policy covering drug production, pricing, distribution, procurement, prescribing, payment, monitoring and evaluation. | Controlled before and after study using data from a nationwide community health services monitoring project. | Percent of prescriptions with an antibiotic | Regulation | National Essential Medicines Program | Education; Environmental Restructuring | Expected | Health Care Worker |
| Lambert MF, et al. Journal of Antimicrobial Chemotherapy. 2007;59(3):537-43. | United Kingdom | Regional | North East Primary Care Trusts | Regional mass media campaign "Antibiotics- tracking down the truth" supported by information materials in GP offices. | Controlled before and after study using data from the prescription pricing authority. | Prescriptions per 1000 STAR-PU | Communication | Public Awareness | Education | Expected | Community |
| Perz JF, Craig AS, Coffey CS, Jorgensen DM, Mitchel E, Hall S, et al. JAMA. 2002;287(23):3103-9. | United States of America | Regional | Knox County Health Department | Regional education campaign to decrease unnecessary antibiotic use in children, including lectures, staff meetings and grand rounds for providers, and pamphlets for parents, and the public along with TV, radio and newspaper coverage. | Controlled before and after study using data from computerized Medicaid files. | Prescription Rate | Communication | Public Awareness | Education | Expected | Community; Health Care Worker |
| Wei X, Yin J, Walley JD, Zhang Z, Hicks JP, Zhou Y, et al. Tropical medicine & international health. 2017;22(9):1166-74. | China | National | Government of China | Chinese National Essential Medicines Policy and zero-markup on prescriptions policy | A controlled pre-post natural experiment analysis using data from the hospital information system. | Outpatient antibiotic prescription rate | Regulation | National Essential Medicines Program | Restriction; Coercion; Environmental Restructuring | Expected | Health Care Worker |
| Yang L, et al. Health Policy and Planning. 2013;28(7):750-60. | China | National | Chinese Ministry of Health, National Development and Reform Commission and seven other agencies | Multifaceted National Chinese Essential Medicines Policy which implemented policies and procedures addressing selection, production, supply and use of essential medicine, pricing, payment, monitoring and evaluation arrangements. | Controlled before and after study using data from health centre databases in Hubei province. | Percentage of prescriptions requiring antibiotics | Regulation | National Essential Medicines Program | Education; Environmental Restructuring | Neutral | Health Care Worker |
| Eythorsson E, et al. BMC Infectious Diseases. 2018;18:10. | Iceland | National | Iceland Department of Health | The 10-valent pneumococcal non-typeable Haemophi- lus influenzae protein D-conjugate vaccine (PHiD-CV10, SynflorixTM) was introduced into the Icelandic paediatric vaccination program without a catch-up for all children born in 2011 and later. | Whole population observational cohort study using data from National Prescription Database | Incidence rates of antimicrobial prescriptions per 100 person-years at risk | Guidelines | Vaccination Guidelines | Environmental restructuring; | Expected | Community |
| Belongia EA, et al. Emerging Infectious Diseases. 2005;11(6):912-20. | United States of America | Regional | Wisconsin Division of Public Health | Establishment of Wisconsin Antibiotic Resistance Network: public and physician educational activities and a media campaign. | Retrospective cohort study using IMS Health data. | Prescription Rate | Communication | Public Awareness | Education | Neutral | Community; Health Care Worker |
| Altunsoy A, et al. International Journal of Medical Sciences. 2011;8(4):339-44. | Turkey | National | Turkish Ministry of Health | Nationwide restriction on use of particular antibiotics without approval by an infectious disease specialist. | Uncontrolled before and after study using hospital pharmacy databases and IMS Health data. | Weight in grams | Regulation | Restricted Use | Restriction | Expected | Health Care Worker |
| Chang SC, et al. Journal of the Formosan Medical Association. 2001;100(3):155-61. | Taiwan | National | National health insurance system in Taiwan | Implementation of a regulation on physician reimbursement where physicians did not receive payment if antibiotic prescriptions did not follow regulations. | Uncontrolled before and after study using data from public health stations. | Prescription Rate | Regulation | Reimbursement Penalty (Physician) | Coercion | Neutral | Health Care Worker |
| Curry M, et al. The New Zealand Medical Journal. 2006;119(1233):U1957. | New Zealand | National | Pharmaceutical Management Agency (PHARMAC) | National public education campaign "Wise Use of Antibiotics Campaign." | Uncontrolled before and after study using data from a telephone questionnaire. | Patient reported antibiotic use | Communication | Public Awareness | Education | Expected | Community |
| Ho M, et al. International Journal of Antimicrobial Agents. 2004;23(5):438-45. | Taiwan | National | Department of Health, Bureau of National Health | Reimbursement regulation forbidding the use of antimicrobials in ambulatory patients with upper respiratory infections (URI) without evidence of bacterial infection. | Uncontrolled before and after study using national health insurance data. | DDD per 1,000 inhabitants per day | Regulation | Restricted Reimbursement | Coercion | Expected | Health Care Worker |
| Kliemann BS, et al. PLoS One. 2016;11(12). | Brazil | National | Government of Brazil | Law abolishing over-the-counter sales of antibiotics in private pharmacies and requiring a copy of the prescription to be retained. | Interrupted time-series analysis using IMS Health data. | DDD per 1000 inhabitants/day | Legislation | Prescription Requirement | Restriction; | Expected | Community |
| Kurt H, et al. Chemotherapy. 2010;56(5):359-63. | Turkey | National | Government of Turkey: Ministries of Health and Finance, SSK, Health Department of the Army, and scientific committees of the Ministry of Health | Restricted reimbursement of antibiotics without prescription or approval by an infectious disease specialist. | Uncontrolled before and after study using IMS Health data. | DDD per 1,000 inhabitants/ day | Regulation | Restricted Reimbursement | Coercion; Restriction | Expected | Health Care Worker |
| Lee YS, et al. Archives of Pharmacal Research. 2014;37(10):1295-300. | South Korea | National | Health Insurance Review and Assessment Services | Introduction of a requirement for public disclosure of antibiotic prescribing rates online. | Uncontrolled before and after study using national health insurance claims data. | DDD per 1000 inhabitants/day | Regulation | Disclosure | Environmental restructuring; | Expected | Health Care Worker |
| Ma XD, et al. BMC Infectious Diseases. 2016;16. | China | National | Chinese Ministry of Health | National policies to improve the intelligent use of antibiotics including restrictions on the type of antibiotics used, and hospital targets for antibiotic use. | Uncontrolled before and after study using hospital data. | DDD per 100 occupied bed days | Regulation | Stewardship | Environmental restructuring; Restriction; | Expected | Health Care Worker |
| MacCara ME, et al. The Annals of pharmacotherapy. 2001;35(7-8):852-8. | Canada | State/  Province | Nova Scotia Department of Health and Wellness | Reimbursement guidelines for the use of fluoroquinolones. | Uncontrolled before and after study using administrative drug claims data. | Number of prescription claims | Regulation | Restricted Reimbursement | Coercion | Expected | Health Care Worker |
| Molstad S, Cars O. Scandinavian Journal of Infectious Diseases. 1999;31(2):191-5. | Sweden | National | Swedish Institute for Infectious Disease Control, Swedish Medical Products Agency, the National Board on Health and Welfare | Development of guidelines for the use of antibiotics in respiratory tract infections, and the development of regional expert groups to provide advice. | Uncontrolled before and after intervention using data from the Nordic Council on Medicines. | DDD per 1,000 inhabitants per day | Guidelines | Committee Development | Education; Environmental Restructuring | Expected | Health Care Worker |
| Park S, et al. Health Policy and Planning. 2005;20(5):302-9. | South Korea | National | Government of Korea | National law prohibiting doctors from dispensing drugs and pharmacists from prescribing drug. | Uncontrolled before and after study using data from the Korean National Health Insurance Claims Database. | Prescription Rate | Legislation | Professional Regulation | Restriction | Expected | Health Care Worker |
| Steffensen FH, et al. Clinical Microbiology and Infection. 1997;3(6):653-7. | Denmark | Regional | National Health Service | Reimbursement for antibiotics was reduced by 50% for subsidized antibiotics and withdrawn for tetracyclines. | Uncontrolled before and after study using data from a pharmacy computerized accounting system. | DDD | Regulation | Reimbursement Penalty (Patient) | Coercion | Expected | Community; Health Care Worker |
| Tao JH, et al. Therapeutic Innovation & Regulatory Science. 2013;47(1):23-31. | China | National | Ministry of Health China | A series of Ministry of Health policies on rational use of antibiotics in China from 2008 to 2011 | Uncontrolled before and after study using data from the Menet Database. | DDD per patient | Regulation | Restricted Use | Restriction; Environmental Restructuring | Expected | Health Care Worker |
| Thornhill MH, et al. BMJ. 2011;342:d2392. | England | National | National Institute for Health and Clinical Excellence | Clinical guidelines for dental and other invasive procedures | Uncontrolled before and after study using data from the NHS prescription pricing division. | Monthly number of prescriptions for antibiotic prophylaxis | Guidelines | Antimicrobial Guidelines | Education | Expected | Health Care Worker |
| Xiao YH, et al. International Journal of Antimicrobial Agents. 2016;48(4):409-14. | China | National | Government of China | Implementation and dissemination of National Essential Drugs List in primary health care settings. | Uncontrolled before and after study using data from health centre records. | Percentage of outpatient prescriptions that contained antibiotics | Regulation | National Essential Medicines Program | Education; Environmental Restructuring | Neutral | Health Care Worker |
| Zhang W, et al. Pharmacoepidemiology and Drug Safety. 2008;17(3):306-11. | China | National | Ministry of Health China | Guidelines for antibacterial use in clinical practice dividing antibiotics into non-restricted use, restricted use and special use categories. | Uncontrolled before and after study using data from hospital databases. | DDD per 100 bed days | Guidelines | Antimicrobial Guidelines | Restriction; Education | Neutral | Health Care Worker |
| Zou XX, et al. Journal of Huazhong University of Science and Technology-Medical Sciences. 2014;34(3):456-63. | China | National | Ministry of Health China | Multifaceted nationwide campaign to overhaul the clinical use of antibiotics dividing antibiotics into non-restricted use, restricted use and special use categories and launching targets for prescribing. | Uncontrolled before and after study using data from hospital administrative and prescription databases. | DDD per 100 inpatient days | Regulation | Restricted Use | Restriction; Education | Expected | Health Care Worker |
| Bavestrello F L, Cabello M A. Revista medica de Chile. 2011;28(2):107-12. | Chile | National | Ministry of Health Chile | Restriction on sale of antibiotics without a prescription in pharmacies. | Descriptive time-series using IMS Health data. | DDD per 1,000 inhabitants per day | Regulation | Prescription Requirement | Education | Expected | Community |
| Bavestrello L, et al. Revista chilena de infectologia. 2002;130(11):1265-72. | Chile | National | Ministry of Health Chile | Restriction on the sale of antibiotics without a prescription in pharmacies, supported by a poster, radio and television campaign. | Uncontrolled before and after study using IMS Health data. | DDD per 1,000 inhabitants per day | Regulation | Prescription Requirement | Restriction; Education | Expected | Community |
| Chahwakilian P, et al. The Journal of Antimicrobial Chemotherapy. 2011;66(12):2872-9. | France | National | French National Health Insurance | National public health campaign “Antibiotics Are Not Automatic!” repeated every winter. | Observational time-series analysis using IMS Health data. | Ambulatory antibiotic prescription rates | Communication | Public Awareness | Education | Expected | Community; Health Care Worker |
| Fan Q, et al. Proceedings of the International Conference on Electronics, Mechanics, Culture and Medicine. 2016;45:654-8. | China | Regional | Chinese Ministry of Health | National policies to improve the intelligent use of antibiotics including the establishment of hospital drug therapeutic committees and targets for antibiotic prescribing. | Retrospective, one group post-test study using data from the hospital information system. | DDD / 100 patient days | Regulation | Stewardship | Environmental restructuring; | Expected | Health Care Worker |
| Furst J, et al. Expert Review of Anti-Infective Therapy. 2015;13(2):279-89. | Slovenia | National | Health Insurance Institute of Slovenia | Suite of education, engineering, economics and enforcement interventions, including workshops, guidelines, prescribing restrictions, and public education. | Uncontrolled before and after study using data from a prescription database. | DDD per 1000 inhabitants/day | Regulation | Restricted Use | Education; Restriction; Persuasion | Expected | Health Care Worker |
| Lambert ML, et al. BMJ Open. 2015;5(2). | Belgium | National | Government of Belgium | Financial and technical support for hiring antimicrobial management teams at acute care hospitals. | Descriptive time-series analysis using hospital administrative data. | DDD per 100 days of hospitalization | Service Provision | Funding | Enablement; Training; | Neutral | Health Care Worker |
| McKay RM et al. Canadian Journal of Infectious Diseases & Medical Microbiology. 2011;22(1):19-24. | Canada | State/  Province | Province of British Columbia Government | Provincial public education campaign "Do Bugs Need Drugs?" including a media campaign, print education material, and educational curricula for school children, parents and older adults in assisted-living facilities. | Descriptive time-series using BC PharmaNet data. | DDD per 1,000 inhabitants per day | Communication | Public Awareness | Education | Expected | Community; Health Care Worker |
| Moura ML, et al. Medicine. 2015;94(38). | Brazil | National | Government of Brazil | Law imposed restricting over-the-counter sales of antimicrobials in pharmacies and requiring a medical prescription for all antimicrobials sales. | Descriptive time-series of pharmacy using IMS Health data. | DDD per 1,000 inhabitants per day | Legislation | Prescription Requirement | Restriction | Expected | Community |
| Shin JY, et al. Pharmacoepidemiology and Drug Safety. 2014;23(12):1320-4. | South Korea | National | Korean Regulatory Agency; Ministry of Food and Drug Safety | Release of guidelines for fluoroquinolone use in patients under 18 through a nationwide computerized drug utilization review system. | Descriptive time-series analysis using data from the Health Insurance Review and Assessment Services National Patients Sample. | Proportion Specific Abx | Guidelines | Antimicrobial Guidelines | Education | Expected | Health Care Worker |
| Long MJ, LaPlant BN, McCormick JC. Journal of the American Pharmacists Association : JAPhA. 2017;57(2):241-7. | USA | National | Federal Bureau of Prisons | A national antimicrobial stewardship program established and developed in the Federal Bureau of Prisons that includes a variety of approaches, including formulary restrictions, clinical guidelines, development of an antimicrobial stewardship group, and dissemination of a tool kit to individual institutions. | Descriptive study using data from electronic medical records. | Rate and Number of prescriptions | Guidelines | Antimicrobial Guidelines | Education; | Expected | Health Care Worker |
| Song SY, et al. Plos One. 2017;12(5). | South Korea | National | Health Insurance Review and Assessment Service | Restrictions on prescribing fluoroquinolones to children under 18. | Descriptive study using data from the Health Insurance Review and Assessment Service. | Annual prescription rate of FQ per 100,000 person-years | Guidelines | Antimicrobial Guidelines | Education; Environmental restructuring | Expected | Health Care Worker |

1. The intended effect column is based upon the authors’ conclusions of the papers and was coded as: expected, neutral, opposite. [↑](#footnote-ref-1)
