# Supplementary material for: Government policy interventions to reduce human antimicrobial use: A systematic review and evidence map
Source: PLoS Med. 2019 Jun 11;16(6):e1002819. doi: 10.1371/journal.pmed.1002819 (PMC6559631; doi:10.1371/journal.pmed.1002819)
Supplement: S1 Text — (DOCX) [file pmed.1002819.s003.docx]

## Search Strategies: Government policy interventions to reduce human antimicrobial use

## MEDLINE SEARCH STRATEGY

1. Anti-Bacterial Agents/

2. Anti-infective Agents/

3. 1 or 2

4. ((antibiotic? or alamethicin? or amdinocillin? or amdinocillin pivoxil? or amikacin? or amoxicillin? or amoxicillin-potassium clavulanate combination? or amphotericin? or ampicillin? or anisomycin? or antimycin? or aurodox? or azithromycin? or azlocillin? or aztreonam? or bacitracin? or bacteriocin? or bambermycin? or bongkrekic acid? or brefeldin? or butirosin sulfate? or calcimycin? or candicidin? or capreomycin? or carbenicillin? or carfecillin? or cefaclor? or cefadroxil? or cefamandole? or cefatrizine? or cefazolin? or cefixime? or cefmenoxime? or cefmetazole? or cefonicid? or cefoperazone? or cefotaxime? or cefotetan? or cefotiam? or cefoxitin? or cefsulodin? or ceftazidime? or ceftizoxime? or ceftriaxone? or cefuroxime? or cephacetrile? or cephalexin? or cephaloglycin? or cephaloridine? or cephalosporin? or cephalothin? or cephamycin? or cephapirin? or cephradine? or chloramphenicol? or chlortetracycline? or citrinin? or clarithromycin? or clavulanic acid? or clavulanic acid? or clindamycin? or cloxacillin? or colistin? or cyclacillin? or dactinomycin? or daptomycin? or demeclocycline? or dibekacin? or dicloxacillin? or dihydrostreptomycin sulfate? or diketopiperazine? or distamycin? or doxycycline? or echinomycin? or edeine? or enviomycin? or erythromycin? or erythromycin estolate? or erythromycin ethylsuccinate? or filipin? or floxacillin? or fluoroquinolone? or fosfomycin? or framycetin? or fusidic acid? or gentamicin? or gramicidin? or hygromycin? or imipenem? or josamycin? or kanamycin? or kitasamycin? or lactam? or lasalocid? or leucomycin? or lincomycin? or lincosamide? or lucensomycin? or lymecycline? or mepartricin? or methacycline? or methicillin? or mezlocillin? or mikamycin? or minocycline? or miocamycin? or moxalactam? or mupirocin? or mycobacillin? or nafcillin? or natamycin? or nebramycin? or neomycin? or netilmicin? or netropsin? or nigericin? or nisin? or norfloxacin? or novobiocin? or nystatin? or ofloxacin? or oleandomycin? or oligomycin? or oxacillin? or oxytetracycline? or paromomycin? or penicillanic acid? or penicillic acid? or penicillin?? or piperacillin? or pivampicillin? or polymyxin b? or polymyxin? or pristinamycin? or prodigiosin? or ribostamycin? or rifabutin? or rifamycin? or ristocetin? or rolitetracycline? or roxarsone? or roxithromycin? or rutamycin? or sirolimu? or sisomicin? or spectinomycin? or spiramycin? or streptogramin?? or streptomycin? or streptovaricin? or sulbactam? or sulbenicillin? or sulfamerazine? or sulfamethoxypyridazine? or talampicillin? or teicoplanin? or tetracycline? or thiamphenicol? or thienamycin? or thiostrepton? or ticarcillin? or tobramycin? or troleandomycin? or tunicamycin? or tylosin? or tyrocidine? or tyrothricin? or valinomycin? or vancomycin? or vernamycin? or viomycin? or virginiamycin? or beta-lactams) adj2 (use* or misuse or consume or consumption or intake or dose or dosage or prescription* or prescrib* or overprescrib* or sale* or rate*)).ab,ti.

5. 3 and 4

6. (adjust* or alter* or change or changes or changing or control* or decreas* or limit* or modify or modified or modifying or reduce or reducing or reduction* or restrict* or appropriate or inappropriate or rational* or irrational*).ab,ti.

7. exp policy/

8. exp policy making/

9. exp legislation as Topic/

10. exp Government/

11. Government Regulation/

12. (program* or campaign* or policy or policies or guideline* or ban or banned or Regulat* or law or laws or prohibit* or restrict* or legislat* or report* or tax* or audit* or formular* or expenditure* or spending or label* or market* or advertis* or consultation*).ab,ti.

13. (health$ adj2 (policy or policies or planning or priorit$)).ab,ti.

14. 7 or 8 or 9 or 10 or 11 or 12 or 13

15. 5 and 6 and 14

16. exp animals/ not humans.sh.

17. 15 not 16

## EMBASE SEARCH STRATEGY

1. exp antiinfective agent/

2. exp antibiotic resistance/

3. ((antibiotic? or alamethicin? or amdinocillin? or amdinocillin pivoxil? or amikacin? or amoxicillin? or amoxicillin-potassium clavulanate combination? or amphotericin? or ampicillin? or anisomycin? or antimycin? or aurodox? or azithromycin? or azlocillin? or aztreonam? or bacitracin? or bacteriocin? or bambermycin? or bongkrekic acid? or brefeldin? or butirosin sulfate? or calcimycin? or candicidin? or capreomycin? or carbenicillin? or carfecillin? or cefaclor? or cefadroxil? or cefamandole? or cefatrizine? or cefazolin? or cefixime? or cefmenoxime? or cefmetazole? or cefonicid? or cefoperazone? or cefotaxime? or cefotetan? or cefotiam? or cefoxitin? or cefsulodin? or ceftazidime? or ceftizoxime? or ceftriaxone? or cefuroxime? or cephacetrile? or cephalexin? or cephaloglycin? or cephaloridine? or cephalosporin? or cephalothin? or cephamycin? or cephapirin? or cephradine? or chloramphenicol? or chlortetracycline? or citrinin? or clarithromycin? or clavulanic acid? or clavulanic acid? or clindamycin? or cloxacillin? or colistin? or cyclacillin? or dactinomycin? or daptomycin? or demeclocycline? or dibekacin? or dicloxacillin? or dihydrostreptomycin sulfate? or diketopiperazine? or distamycin? or doxycycline? or echinomycin? or edeine? or enviomycin? or erythromycin? or erythromycin estolate? or erythromycin ethylsuccinate? or filipin? or floxacillin? or fluoroquinolone? or fosfomycin? or framycetin? or fusidic acid? or gentamicin? or gramicidin? or hygromycin? or imipenem? or josamycin? or kanamycin? or kitasamycin? or lactam? or lasalocid? or leucomycin? or lincomycin? or lincosamide? or lucensomycin? or lymecycline? or mepartricin? or methacycline? or methicillin? or mezlocillin? or mikamycin? or minocycline? or miocamycin? or moxalactam? or mupirocin? or mycobacillin? or nafcillin? or natamycin? or nebramycin? or neomycin? or netilmicin? or netropsin? or nigericin? or nisin? or norfloxacin? or novobiocin? or nystatin? or ofloxacin? or oleandomycin? or oligomycin? or oxacillin? or oxytetracycline? or paromomycin? or penicillanic acid? or penicillic acid? or penicillin?? or piperacillin? or pivampicillin? or polymyxin b? or polymyxin? or pristinamycin? or prodigiosin? or ribostamycin? or rifabutin? or rifamycin? or ristocetin? or rolitetracycline? or roxarsone? or roxithromycin? or rutamycin? or sirolimu? or sisomicin? or spectinomycin? or spiramycin? or streptogramin?? or streptomycin? or streptovaricin? or sulbactam? or sulbenicillin? or sulfamerazine? or sulfamethoxypyridazine? or talampicillin? or teicoplanin? or tetracycline? or thiamphenicol? or thienamycin? or thiostrepton? or ticarcillin? or tobramycin? or troleandomycin? or tunicamycin? or tylosin? or tyrocidine? or tyrothricin? or valinomycin? or vancomycin? or vernamycin? or viomycin? or virginiamycin? or beta-lactams) adj2 (use* or misuse or consume or consumption or intake or dose or dosage or prescription* or prescrib* or overprescrib* or sale* or rate*)).ab,ti.

4. 1 or 2

5. 3 and 4

6. (adjust* or alter* or change or changes or changing or control* or decreas* or limit* or modify or modified or modifying or reduce or reducing or reduction* or restrict* or appropriate or inappropriate or rational* or irrational*).ab,ti.

7. exp policy/

8. exp health care policy/

9. exp legislation, drug/

10. (program* or campaign* or policy or policies or guideline* or ban or banned or Regulat* or law or laws or prohibit* or restrict* or legislat* or public or report* or tax* or audit* or formular* or feedback).ab,ti.

11. (health$ adj3 (policy or policies or planning or priorit$)).ab,ti.

12. 7 or 8 or 9 or 10 or 11

13. 5 and 6 and 12

14. exp animals/ not humans.sh.

15. 13 not 14

## WEB OF SCIENCE SEARCH STRATEGY

1) TS=(antibiotic* near/2 use*) OR

TS=(antibiotic* near/2 misuse) OR

TS=(antibiotic* near/2 consume) OR

TS=(antibiotic* near/2 consumption) OR

TS=(antibiotic* near/2 intake) OR

TS=(antibiotic* near/2 dose) OR

TS=(antibiotic* near/2 dosage) OR

TS=(antibiotic* near/2 prescription*) OR

TS=(antibiotic* near/2 prescrib*) OR

TS=(antibiotic* near/2 overprescrib*) OR

TS=(antibiotic* near/2 sale*) OR

TS=(antibiotic* near/2 rate*) OR

TS=(antimicrobial* near/2 use*) OR

TS=(antimicrobial* near/2 misuse) OR

TS=(antimicrobial* near/2 consume) OR

TS=(antimicrobial* near/2 consumption) OR

TS=(antimicrobial* near/2 intake) OR

TS=(antimicrobial* near/2 dose) OR

TS=(antimicrobial* near/2 dosage) OR

TS=(antimicrobial* near/2 prescription*) OR

TS=(antimicrobial* near/2 prescrib*) OR

TS=(antimicrobial* near/2 overprescrib*) OR

TS=(antimicrobial* near/2 sale*) OR

TS=(antimicrobial* near/2 rate*)

2) TS=(adjust*) OR TS=(alter*) OR TS=(change) OR TS=(changes) OR TS=(changing) OR TS=(control*) OR TS=(decreas*) OR TS=(limit*) OR TS=(modify) OR TS=(modified) OR TS=(modifying) OR TS=(reduce) OR TS=(reducing) OR TS=(reduction*) OR TS=(restrict*) OR TS=(appropriate) OR TS=(inappropriate) OR TS=(rational*) OR TS=(irrational*)

3) TS=(program* OR campaign* OR policy OR policies OR guideline* OR ban OR banned OR regulat* OR law OR laws OR prohibit* OR restrict* OR legislat* OR report* OR tax* or audit* OR formular* or expenditure* or spending or label* or maket* or advertis* or consultation*) OR TS=(health* NEAR/2 policy) OR TS=(health* NEAR/2 policies) OR TS=(health* NEAR/2 planning) OR TS=(health* NEAR2 priorit*)

4)#1 AND #2 AND #3

5) TS=(resistan*) OR TS=(steward*)

6) #4 and #5

7) TS=( *food* OR aqua* OR animal* OR vetrinar* OR livestock OR "live stock" OR agricultur*)

8) #6 NOT #7

## CENTRAL SEARCH STRATEGY

1. MeSH descriptor: [Anti-Infective Agents] explode all trees
2. ((antibiotic? or alamethicin? or amdinocillin? or amdinocillin pivoxil? or amikacin? or amoxicillin? or amoxicillin-potassium clavulanate combination? or amphotericin? or ampicillin? or anisomycin? or antimycin? or aurodox? or azithromycin? or azlocillin? or aztreonam? or bacitracin? or bacteriocin? or bambermycin? or bongkrekic acid? or brefeldin? or butirosin sulfate? or calcimycin? or candicidin? or capreomycin? or carbenicillin? or carfecillin? or cefaclor? or cefadroxil? or cefamandole? or cefatrizine? or cefazolin? or cefixime? or cefmenoxime? or cefmetazole? or cefonicid? or cefoperazone? or cefotaxime? or cefotetan? or cefotiam? or cefoxitin? or cefsulodin? or ceftazidime? or ceftizoxime? or ceftriaxone? or cefuroxime? or cephacetrile? or cephalexin? or cephaloglycin? or cephaloridine? or cephalosporin? or cephalothin? or cephamycin? or cephapirin? or cephradine? or chloramphenicol? or chlortetracycline? or citrinin? or clarithromycin? or clavulanic acid? or clavulanic acid? or clindamycin? or cloxacillin? or colistin? or cyclacillin? or dactinomycin? or daptomycin? or demeclocycline? or dibekacin? or dicloxacillin? or dihydrostreptomycin sulfate? or diketopiperazine? or distamycin? or doxycycline? or echinomycin? or edeine? or enviomycin? or erythromycin? or erythromycin estolate? or erythromycin ethylsuccinate? or filipin? or floxacillin? or fluoroquinolone? or fosfomycin? or framycetin? or fusidic acid? or gentamicin? or gramicidin? or hygromycin? or imipenem? or josamycin? or kanamycin? or kitasamycin? or lactam? or lasalocid? or leucomycin? or lincomycin? or lincosamide? or lucensomycin? or lymecycline? or mepartricin? or methacycline? or methicillin? or mezlocillin? or mikamycin? or minocycline? or miocamycin? or moxalactam? or mupirocin? or mycobacillin? or nafcillin? or natamycin? or nebramycin? or neomycin? or netilmicin? or netropsin? or nigericin? or nisin? or norfloxacin? or novobiocin? or nystatin? or ofloxacin? or oleandomycin? or oligomycin? or oxacillin? or oxytetracycline? or paromomycin? or penicillanic acid? or penicillic acid? or penicillin?? or piperacillin? or pivampicillin? or polymyxin b? or polymyxin? or pristinamycin? or prodigiosin? or ribostamycin? or rifabutin? or rifamycin? or ristocetin? or rolitetracycline? or roxarsone? or roxithromycin? or rutamycin? or sirolimu? or sisomicin? or spectinomycin? or spiramycin? or streptogramin?? or streptomycin? or streptovaricin? or sulbactam? or sulbenicillin? or sulfamerazine? or sulfamethoxypyridazine? or talampicillin? or teicoplanin? or tetracycline? or thiamphenicol? or thienamycin? or thiostrepton? or ticarcillin? or tobramycin? or troleandomycin? or tunicamycin? or tylosin? or tyrocidine? or tyrothricin? or valinomycin? or vancomycin? or vernamycin? or viomycin? or virginiamycin? or beta-lactams) adj2 (use* or misuse or consume or consumption or intake or dose or dosage or prescription* or prescrib* or overprescrib* or sale* or rate*))
3. #1 and #2
4. (adjust* or alter* or change or changes or changing or control* or decreas* or limit* or modify or modified or modifying or reduce or reducing or reduction* or restrict* or appropriate or inappropriate or rational* or irrational*)
5. MeSH descriptor: [Policy] explode all trees
6. MeSH descriptor: [Legislation as Topic] explode all trees
7. MeSH descriptor: [Government] explode all trees
8. MeSH descriptor: [Government Regulation] explode all trees
9. MeSH descriptor: [Policy Making] explode all trees
10. (program* or campaign* or policy or policies or guideline* or ban or banned or Regulat* or law or laws or prohibit* or restrict* or legislat* or report* or tax* or audit* or formular* or expenditure* or spending or label* or market* or advertis* or consultation*)
11. (health$ near/2 (policy or policies or planning or priorit$))
12. #5 or #6 or #7 or #8 or #9 or #10 or #11
13. #3 and #4 and #12

## CINAHL SEARCH STRATEGY

## (MH "Antiinfective Agents+")

1. (TI antibiotic* N2 use*) OR (AB antibiotic* N2 use*) OR (HW antibiotic* N2 use*) OR (TI antibiotic* N2 misuse) OR (AB antibiotic* N2 misuse) OR (HW antibiotic* N2 misuse) OR (TI antibiotic* N2 consume ) OR (AB antibiotic* N2 consume ) OR (HW antibiotic* N2 consume ) OR (TI antibiotic* N2 consumption) OR (AB antibiotic* N2 consumption) OR (HW antibiotic* N2 consumption) OR (TI antibiotic* N2 intake ) OR (AB antibiotic* N2 intake ) OR (HW antibiotic* N2 intake ) OR (TI antibiotic* N2 dose ) OR (AB antibiotic* N2 dose ) OR (HW antibiotic* N2 dose ) OR (TI antibiotic* N2 dosage ) OR (AB antibiotic* N2 dosage ) OR (HW antibiotic* N2 dosage) OR (TI antibiotic* N2 prescription* ) OR (AB antibiotic* N2 prescription* ) OR (HW antibiotic* N2 prescription* ) OR (TI antibiotic* N2 prescrib* ) OR (AB antibiotic* N2 prescrib* ) OR (HW antibiotic* N2 prescrib* ) OR (TI antibiotic* N2 overprescrib* ) OR (AB antibiotic* N2 overprescrib* ) OR (HW antibiotic* N2 overprescrib* ) OR (TI antibiotic* N2 sale* ) OR (AB antibiotic* N2 sale* ) OR (HW antibiotic* N2 sale* )
2. (TI antibiotic* N2 rate* ) OR (AB antibiotic* N2 rate* ) OR (HW antibiotic* N2 rate* ) OR (TI antimicrobial* N2 use*) OR (AB antimicrobial* N2 use*) OR (HW antimicrobial* N2 use*)
3. (TI antimicrobial* N2 misuse) OR (AB antimicrobial* N2 misuse) OR (HW antimicrobial* N2 misuse) OR (TI antimicrobial* N2 consume ) OR (AB antimicrobial* N2 consume ) OR (HW antimicrobial* N2 consume ) OR (TI antimicrobial* N2 consumption) OR (AB antimicrobial* N2 consumption) OR (HW antimicrobial* N2 consumption) OR (TI antimicrobial* N2 intake ) OR (AB antimicrobial* N2 intake ) OR (HW antimicrobial* N2 intake ) OR (TI antimicrobial* N2 dose ) OR (AB antimicrobial* N2 dose ) OR (HW antimicrobial* N2 dose ) OR (TI antimicrobial* N2 dosage ) OR (AB antimicrobial* N2 dosage ) OR (HW antimicrobial* N2 dosage ) OR (TI antimicrobial* N2 prescription* ) OR (AB antimicrobial* N2 prescription* ) OR (HW antimicrobial* N2 prescription* ) OR (TI antimicrobial* N2 prescrib* ) OR (AB antimicrobial* N2 prescrib* ) OR (HW antimicrobial* N2 prescrib* ) OR (TI antimicrobial* N2 overprescrib* ) OR (AB antimicrobial* N2 overprescrib* ) OR (HW antimicrobial* N2 overprescrib* ) OR (TI antimicrobial* N2 sale* ) OR (AB antimicrobial* N2 sale* ) OR (HW antimicrobial* N2 sale* ) OR (TI antimicrobial* N2 rate* ) OR (AB antimicrobial* N2 rate* ) OR (HW antimicrobial* N2 rate* )
4. (TI adjust* ) OR (AB adjust* ) OR (TI alter* ) OR (AB alter* ) OR (TI change ) OR (AB change ) OR (TI changes ) OR (AB changes ) OR (TI changing ) OR (AB changing ) OR (TI control* ) OR (AB control* ) OR (TI decreas* ) OR (AB decreas* ) OR (TI limit* ) OR (AB limit* ) OR (TI modify ) OR (AB modify ) OR (TI modified ) OR (AB modified ) OR (TI modifying ) OR (AB modifying ) OR (TI reduce ) OR (AB reduce ) OR (TI reducing ) OR (AB reducing ) OR (TI reduction* ) OR (AB reduction* ) OR (TI restrict* ) OR (AB restrict* ) OR (TI appropriate ) OR (AB appropriate ) OR (TI inappropriate ) OR (AB inappropriate ) OR (TI rational* ) OR (AB rational* ) OR (TI irrational* ) OR (AB irrational* )
5. (MH "Hospital Policies")
6. (MH "Health Policy Studies")
7. (MH "Policy Studies")
8. (MH "Public Policy+")
9. (MH "Policy Making")
10. (MH "Legislation, Drug")
11. (TI program* ) OR (AB program* ) OR (TI campaign* ) OR (AB campaign* ) OR (TI policy ) OR (AB policy ) OR (TI policies ) OR (AB policies ) OR (TI guideline* ) OR (AB guideline* ) OR (TI ban ) OR (AB ban ) OR (TI banned ) OR (AB banned ) OR (TI Regulat* ) OR (AB Regulat* ) OR (TI law ) OR (AB law ) OR (TI laws ) OR (AB laws ) OR (TI prohibit* ) OR (AB prohibit* ) OR (TI restrict* ) OR (AB restrict* ) OR (TI legislat* ) OR (AB legislat* ) OR (TI report* ) OR (AB report* ) OR (TI tax* ) OR (AB tax* )  OR (TI audit* ) OR (AB audit* ) OR (TI formular* ) OR (AB formular* ) OR (TI expenditure* ) OR (AB expenditure* ) OR (TI spending ) OR (AB spending ) OR (TI label* ) OR (AB label* ) OR (TI market* ) OR (AB market* ) OR (TI advertis* ) OR (AB advertis* ) OR (TI consultation* ) OR (AB consultation* ) OR (TI health* N2 policy) OR (AB health* N2 use*) OR (TI health* N2 policies ) OR (AB health* N2 policies ) OR (TI health* N2 planning ) OR (AB health* N2 planning ) OR (TI health* N2 priorit* ) OR (AB health* N2 priorit* )
12. 12 AND (5 OR 6 OR 7 OR 8 OR 9 OR 10 OR 11)) AND (1 AND (2 OR 3 OR 4))

## PAIS INDEX

1. (ti(((antibiotic* OR antimicrobial*) NEAR/2 (use* OR misuse OR consume OR consumption OR intake OR dose OR dosage OR prescription* OR prescrib* OR overprescrib* OR sale* OR rate*))) OR ab(((antibiotic* OR antimicrobial*) NEAR/2 (use* OR misuse OR consume OR consumption OR intake OR dose OR dosage OR prescription* OR prescrib* OR overprescrib* OR sale* OR rate*))))
2. (ti(adjust* or alter* or change or changes or changing or control* or decreas* or limit* or modify or modified or modifying or reduce or reducing or reduction* or restrict* or appropriate or inappropriate or rational* or irrational*) OR ab(adjust* or alter* or change or changes or changing or control* or decreas* or limit* or modify or modified or modifying or reduce or reducing or reduction* or restrict* or appropriate or inappropriate or rational* or irrational*))
3. (ti(program* or campaign* or policy or policies or guideline* or ban or banned or Regulat* or law or laws or prohibit* or restrict* or legislat* or report* or tax* or audit* or formular* or expenditure* or spending or label* or market* or advertis* of consultation*) OR ab(program* or campaign* or policy or policies or guideline* or ban or banned or Regulat* or law or laws or prohibit* or restrict* or legislat* or public or report* or tax* or audit* or formular* orexpenditure* or spending or label* or market* or advertis* of consultation*)) OR (ti(health* NEAR/2 (policy or policies or planning or priorit*)) or ab(health* NEAR/2 (policy or policies or planning or priorit*)))
4. 1 AND 2 AND 3

## MEDLINE NOT PUBMED

 ((((((((((Anti-Bacterial Agents[MeSH Terms]) OR Anti-infective Agents[MeSH Terms])) AND ((antibiotic?[Title/Abstract] OR alamethicin?[Title/Abstract] OR amdinocillin?[Title/Abstract] OR amdinocillin pivoxil?[Title/Abstract] OR amikacin?[Title/Abstract] OR amoxicillin?[Title/Abstract] OR amoxicillin-potassium clavulanate combination?[Title/Abstract] OR amphotericin?[Title/Abstract] OR ampicillin?[Title/Abstract] OR anisomycin?[Title/Abstract] OR antimycin?[Title/Abstract] OR aurodox?[Title/Abstract] OR azithromycin?[Title/Abstract] OR azlocillin?[Title/Abstract] OR aztreonam?[Title/Abstract] OR bacitracin?[Title/Abstract] OR bacteriocin?[Title/Abstract] OR bambermycin?[Title/Abstract] OR bongkrekic acid?[Title/Abstract] OR brefeldin?[Title/Abstract] OR butirosin sulfate?[Title/Abstract] OR calcimycin?[Title/Abstract] OR candicidin?[Title/Abstract] OR capreomycin?[Title/Abstract] OR carbenicillin?[Title/Abstract] OR carfecillin?[Title/Abstract] OR cefaclor?[Title/Abstract] OR cefadroxil?[Title/Abstract] OR cefamandole?[Title/Abstract] OR cefatrizine?[Title/Abstract] OR cefazolin?[Title/Abstract] OR cefixime?[Title/Abstract] OR cefmenoxime?[Title/Abstract] OR cefmetazole?[Title/Abstract] OR cefonicid?[Title/Abstract] OR cefoperazone?[Title/Abstract] OR cefotaxime?[Title/Abstract] OR cefotetan?[Title/Abstract] OR cefotiam?[Title/Abstract] OR cefoxitin?[Title/Abstract] OR cefsulodin?[Title/Abstract] OR ceftazidime?[Title/Abstract] OR ceftizoxime?[Title/Abstract] OR ceftriaxone?[Title/Abstract] OR cefuroxime?[Title/Abstract] OR cephacetrile?[Title/Abstract] OR cephalexin?[Title/Abstract] OR cephaloglycin?[Title/Abstract] OR cephaloridine?[Title/Abstract] OR cephalosporin?[Title/Abstract] OR cephalothin?[Title/Abstract] OR cephamycin?[Title/Abstract] OR cephapirin?[Title/Abstract] OR cephradine?[Title/Abstract] OR chloramphenicol?[Title/Abstract] OR chlortetracycline?[Title/Abstract] OR citrinin?[Title/Abstract] OR clarithromycin?[Title/Abstract] OR clavulanic acid?[Title/Abstract] OR clavulanic acid?[Title/Abstract] OR clindamycin?[Title/Abstract] OR cloxacillin?[Title/Abstract] OR colistin?[Title/Abstract] OR cyclacillin?[Title/Abstract] OR dactinomycin?[Title/Abstract] OR daptomycin?[Title/Abstract] OR demeclocycline?[Title/Abstract] OR dibekacin?[Title/Abstract] OR dicloxacillin?[Title/Abstract] OR dihydrostreptomycin sulfate?[Title/Abstract] OR diketopiperazine?[Title/Abstract] OR distamycin?[Title/Abstract] OR doxycycline?[Title/Abstract] OR echinomycin?[Title/Abstract] OR edeine?[Title/Abstract] OR enviomycin?[Title/Abstract] OR erythromycin?[Title/Abstract] OR erythromycin estolate?[Title/Abstract] OR erythromycin ethylsuccinate?[Title/Abstract] OR filipin?[Title/Abstract] OR floxacillin?[Title/Abstract] OR fluoroquinolone?[Title/Abstract] OR fosfomycin?[Title/Abstract] OR framycetin?[Title/Abstract] OR fusidic acid?[Title/Abstract] OR gentamicin?[Title/Abstract] OR gramicidin?[Title/Abstract] OR hygromycin?[Title/Abstract] OR imipenem?[Title/Abstract] OR josamycin?[Title/Abstract] OR kanamycin?[Title/Abstract] OR kitasamycin?[Title/Abstract] OR lactam?[Title/Abstract] OR lasalocid?[Title/Abstract] OR leucomycin?[Title/Abstract] OR lincomycin?[Title/Abstract] OR lincosamide?[Title/Abstract] OR lucensomycin?[Title/Abstract] OR lymecycline?[Title/Abstract] OR mepartricin?[Title/Abstract] OR methacycline?[Title/Abstract] OR methicillin?[Title/Abstract] OR mezlocillin?[Title/Abstract] OR mikamycin?[Title/Abstract] OR minocycline?[Title/Abstract] OR miocamycin?[Title/Abstract] OR moxalactam?[Title/Abstract] OR mupirocin?[Title/Abstract] OR mycobacillin?[Title/Abstract] OR nafcillin?[Title/Abstract] OR natamycin?[Title/Abstract] OR nebramycin?[Title/Abstract] OR neomycin?[Title/Abstract] OR netilmicin?[Title/Abstract] OR netropsin?[Title/Abstract] OR nigericin?[Title/Abstract] OR nisin?[Title/Abstract] OR norfloxacin?[Title/Abstract] OR novobiocin?[Title/Abstract] OR nystatin?[Title/Abstract] OR ofloxacin?[Title/Abstract] OR oleandomycin?[Title/Abstract] OR oligomycin?[Title/Abstract] OR oxacillin?[Title/Abstract] OR oxytetracycline?[Title/Abstract] OR paromomycin?[Title/Abstract] OR penicillanic acid?[Title/Abstract] OR penicillic acid?[Title/Abstract] OR penicillin??[Title/Abstract] OR piperacillin?[Title/Abstract] OR pivampicillin?[Title/Abstract] OR polymyxin b?[Title/Abstract] OR polymyxin?[Title/Abstract] OR pristinamycin?[Title/Abstract] OR prodigiosin?[Title/Abstract] OR ribostamycin?[Title/Abstract] OR rifabutin?[Title/Abstract] OR rifamycin?[Title/Abstract] OR ristocetin?[Title/Abstract] OR rolitetracycline?[Title/Abstract] OR roxarsone?[Title/Abstract] OR roxithromycin?[Title/Abstract] OR rutamycin?[Title/Abstract] OR sirolimu?[Title/Abstract] OR sisomicin?[Title/Abstract] OR spectinomycin?[Title/Abstract] OR spiramycin?[Title/Abstract] OR streptogramin??[Title/Abstract] OR streptomycin?[Title/Abstract] OR streptovaricin?[Title/Abstract] OR sulbactam?[Title/Abstract] OR sulbenicillin?[Title/Abstract] OR sulfamerazine?[Title/Abstract] OR sulfamethoxypyridazine?[Title/Abstract] OR talampicillin?[Title/Abstract] OR teicoplanin?[Title/Abstract] OR tetracycline?[Title/Abstract] OR thiamphenicol?[Title/Abstract] OR thienamycin?[Title/Abstract] OR thiostrepton?[Title/Abstract] OR ticarcillin?[Title/Abstract] OR tobramycin?[Title/Abstract] OR troleandomycin?[Title/Abstract] OR tunicamycin?[Title/Abstract] OR tylosin?[Title/Abstract] OR tyrocidine?[Title/Abstract] OR tyrothricin?[Title/Abstract] OR valinomycin?[Title/Abstract] OR vancomycin?[Title/Abstract] OR vernamycin?[Title/Abstract] OR viomycin?[Title/Abstract] OR virginiamycin?[Title/Abstract] OR beta-lactams[Title/Abstract]))) AND ((use*[Title/Abstract] OR misuse[Title/Abstract] OR consume[Title/Abstract] OR consumption[Title/Abstract] OR intake[Title/Abstract] OR dose[Title/Abstract] OR dosage[Title/Abstract] OR prescription*[Title/Abstract] OR prescrib*[Title/Abstract] OR overprescrib*[Title/Abstract] OR sale*[Title/Abstract] OR rate*[Title/Abstract])))) AND ((adjust*[Title/Abstract] OR alter*[Title/Abstract] OR change[Title/Abstract] OR changes[Title/Abstract] OR changing[Title/Abstract] OR control*[Title/Abstract] OR decreas*[Title/Abstract] OR limit*[Title/Abstract] OR modify[Title/Abstract] OR modified[Title/Abstract] OR modifying[Title/Abstract] OR reduce[Title/Abstract] OR reducing[Title/Abstract] OR reduction*[Title/Abstract] OR restrict*[Title/Abstract] OR appropriate[Title/Abstract] OR inappropriate[Title/Abstract] OR rational*[Title/Abstract] OR irrational*[Title/Abstract]))) AND ((((((policy[MeSH Terms]) OR policy making[MeSH Terms]) OR legislation as Topic[MeSH Terms]) OR Government[MeSH Terms]) OR Government Regulation[MeSH Terms]) OR (program*[Title/Abstract] OR campaign*[Title/Abstract] OR policy[Title/Abstract] OR policies[Title/Abstract] OR guideline*[Title/Abstract] OR ban[Title/Abstract] OR banned[Title/Abstract] OR Regulat*[Title/Abstract] OR law[Title/Abstract] OR laws[Title/Abstract] OR prohibit*[Title/Abstract] OR restrict*[Title/Abstract] OR legislat*[Title/Abstract] OR report*[Title/Abstract] OR tax*[Title/Abstract] OR audit*[Title/Abstract] OR formular*[Title/Abstract] OR expenditure*[Title/Abstract] OR spending[Title/Abstract] OR label*[Title/Abstract] OR market*[Title/Abstract] OR advertis*[Title/Abstract] OR consultation*[Title/Abstract])))) AND (in process[sb] OR medline[sb])) AND 2019/01/27"[Entrez Date]:"3000"[Entrez Date]) Schema: all Schema: all
