# Supplementary material for: Government policy interventions to reduce human antimicrobial use: A systematic review and evidence map
Source: PLoS Med. 2019 Jun 11;16(6):e1002819. doi: 10.1371/journal.pmed.1002819 (PMC6559631; doi:10.1371/journal.pmed.1002819)
Supplement: S2 Text — (DOCX) [file pmed.1002819.s004.docx]

AMU Policies - Data Extraction Tool

## General Information

| First Author Name | |  |
| --- | --- | --- |
| Publication Year | |  |
| Journal Name | |  |
| Contact Details | Name |  |
|  | Affiliation |  |
|  | Email Address |  |
| Publication Type | | CHOOSE:  **Research Study**  **Abstract**  **Grey Literature** |
| Ethics Approval Received? | |  |
| Funding Source | |  |
| Country of Implementation | |  |

## PICO Summary

| Population |  |
| --- | --- |
| Intervention |  |
| Comparator |  |
| Outcome |  |

## CONTINUE?

| Does the study meet the PICO requirements? | **YES / NO** |
| --- | --- |
| Rationale for excluding at this stage |  |

## **If yes, continue to next section; if no, stop.**

## Study Characteristics

|  | | Description | Page # |
| --- | --- | --- | --- |
| Intervention Aim | |  |  |
| Study Aim | |  |  |
| Type of study |  | | |
| Study Timing | |  |  |
| Population of interest | |  |  |
| Data Source | Source |  |  |
|  | Description of data |  |  |
| Inclusion Criteria | |  |  |
| Exclusion Criteria | |  |  |
| Outcome measure | |  |  |
| Sample Size | N of Participants |  |  |
|  | Sample size calculation |  |  |
|  | Power calculation |  |  |
| Method of recruitment | |  |  |
| Randomization/Allocation Methods | |  |  |
| Notes |  | | |

## Intervention Characteristics

|  |  | Description | Page # |
| --- | --- | --- | --- |
| Enacting Government | |  |  |
| Description of Policy Intervention | |  |  |
| Description of Intervention Groups | |  |  |
| Number of Intervention Groups | |  |  |
| Intervention Dates | |  |  |
| Intervention Duration | Duration: Pre-Intervention |  |  |
|  | N of Pre-intervention timepoints |  |  |
|  | Duration: Follow-up |  |  |
|  | N of follow-up timepoints |  |  |
| Setting | |  |  |
| Delivery (eg. Stages (sequential or simultaneous), timing, frequency, duration, intensity, fidelity – process indicators) | |  |  |
| Notes | |  | |

## Results Summary

|  | Description | Page |
| --- | --- | --- |
| Main findings |  |  |
| Secondary Findings |  |  |
| Key conclusions of study authors |  |  |
| Notes |  | |

| Does the study design meet the inclusion criteria for the Effectiveness Review? |  |
| --- | --- |
